# Association Between Metabolic Dysfunction-Associated Steatotic Liver Disease and Sarcopenia: A Systematic Review and Meta-analysis

**DOI:** 10.7759/cureus.94766

**Published:** 2025-10-17

**Authors:** Helai Hussaini, Fikadu Woreta, Olga Sarah, Olaniyi Fadeyi, Rahman Hameed Mohammed Abdul, Sonalben Chaudhary, Mohammed Qasim Rauf, Shamsha Hirani

**Affiliations:** 1 Internal Medicine, West Anaheim Medical Center, Anaheim, USA; 2 Internal Medicine, Inova, Silver Spring, USA; 3 Medicine, Tartus University, Tartus, SYR; 4 Gastroenterology and Hepatology, Royal Derby Hospital, Stoke-on-Trent, GBR; 5 Internal Medicine, Zydus Sitapur Hospital, Sitapur, IND; 6 Trauma and Orthopaedics, The Hillingdon Hospitals NHS Foundation Trust, London, GBR; 7 Cardiology, Baqai Hospital, Karachi, PAK

**Keywords:** liver disease, meta-analysis, metabolic dysfunction-associated steatotic liver disease, muscle mass, sarcopenia

## Abstract

This systematic review and meta-analysis evaluated the prevalence of sarcopenia in patients with metabolic dysfunction-associated steatotic liver disease (MASLD) compared to controls. A comprehensive search across PubMed, Embase, Cochrane Library, and Web of Science databases was conducted from January 2015 to August 2025, identifying studies that compared the prevalence of sarcopenia between MASLD patients and non-MASLD controls. Sixteen studies comprising diverse populations from Korea, China, the United States, Italy, the Netherlands, and the United Kingdom were included, with sample sizes ranging from 57 to 18,815 participants. Sarcopenia assessment methods varied across studies, including dual-energy X-ray absorptiometry (DXA), bioelectrical impedance analysis (BIA), computed tomography (CT), and functional tests. Statistical analyses were performed using RevMan 5.4 (The Cochrane Collaboration, London, England, UK) with random-effects models to calculate pooled odds ratios (ORs). The meta-analysis revealed a significantly higher prevalence of sarcopenia in MASLD patients (14.86%) compared to controls (6.49%), with a pooled OR of 2.24 (95% CI: 1.74-2.89, p < 0.001). Substantial heterogeneity was observed (I² = 95%) across studies. Subgroup analyses demonstrated stronger associations in cohort studies versus cross-sectional studies, in populations under 50 years of age, and in studies from Korea and the United States. The bidirectional relationship between MASLD and sarcopenia is supported by shared pathophysiological mechanisms, including insulin resistance, chronic inflammation, and altered protein metabolism. These findings suggest that MASLD patients should undergo routine sarcopenia screening, and interventions targeting muscle health may benefit both conditions. The substantial burden of sarcopenia in MASLD patients highlights the need for integrated management approaches addressing both hepatic and muscular manifestations.

## Introduction and background

Metabolic dysfunction-associated steatotic liver disease (MASLD) has recently been proposed as a modern conceptual framework to replace the conventional term non-alcoholic fatty liver disease (NAFLD) [1]. This update, introduced by leading international experts in 2023, aims to better capture the intricate metabolic disturbances and multifactorial pathogenesis underlying steatotic liver disease (SLD) [1]. Traditionally, NAFLD was used to identify SLD in individuals who consume little or no alcohol and present with liver inflammation resembling alcoholic steatohepatitis. The term encompassed the entire spectrum of the disease, including simple hepatic steatosis, non-alcoholic steatohepatitis (steatosis accompanied by inflammation), and progressive stages such as fibrosis and cirrhosis [2].

The diagnosis of NAFLD has traditionally relied on a process of exclusion, whereby other liver conditions, such as viral hepatitis, autoimmune liver disease, or alcoholic liver injury, are ruled out before assessing SLD progression [3]. Although individuals with both metabolic risk factors and alcohol consumption require timely intervention, no dedicated diagnostic category has previously existed for this population. In contrast, the MASLD framework introduces positive diagnostic criteria that specifically identify SLD linked to metabolic dysfunction, rather than relying on exclusion [1]. This shift emphasizes the central role of metabolic factors in SLD, recognizing that the negative clinical outcomes associated with NAFLD are largely driven by metabolic syndrome [4]. Current projections indicate a concerning epidemiological trajectory, with MASLD prevalence expected to exceed 55% among US adults by 2040, driven by rising obesity and type 2 diabetes rates [5]. Particularly alarming is the disease burden among individuals with diabetes, where MASLD affects approximately two-thirds of patients with type 2 diabetes, with 15%-38% developing metabolic dysfunction-associated steatohepatitis (MASH) with clinically significant liver fibrosis [6]. Beyond hepatic manifestations, MASLD is associated with increased risks of cardiovascular disease (43% increase), chronic kidney disease (38%), and extrahepatic cancers (54%) and represents a multisystemic disorder with far-reaching health implications [7].

Sarcopenia, defined as the progressive and generalized loss of skeletal muscle mass, strength, and function, has gained recognition as a critical extrahepatic manifestation of MASLD [8,9]. This age-related syndrome affects up to 27% of individuals aged 60 years and older, significantly impacting quality of life and increasing mortality risk [10]. The pathophysiological relationship between MASLD and sarcopenia is complex and bidirectional, sharing common underlying mechanisms including insulin resistance, chronic inflammation, oxidative stress, and dysregulated protein synthesis [11]. The liver plays a crucial role in protein metabolism and synthesis of branched-chain amino acids, while skeletal muscle serves as the primary site for amino acid metabolism and glucose homeostasis [12].

Emerging evidence suggests that sarcopenia may not only be a consequence of MASLD progression but also a contributing factor to disease development and severity. Recent meta-analyses have demonstrated that patients with MASLD exhibit a significantly higher prevalence of sarcopenia compared to healthy controls, with odds ratios (ORs) ranging from 1.25 to 2.08 [13,14]. A recent systematic review reported a pooled sarcopenia prevalence of 23.5% among MASLD patients, with notable regional variations and differences based on diagnostic criteria and measurement methods [15].

Given the substantial burden of both MASLD and sarcopenia on global health systems and their complex bidirectional relationship, there is an urgent need for a comprehensive synthesis of current evidence. Understanding the true prevalence of sarcopenia in MASLD patients and its clinical implications is essential for developing targeted screening strategies, prevention programs, and therapeutic interventions. Therefore, this systematic review and meta-analysis aims to provide a comprehensive evaluation of the prevalence and effects of sarcopenia in patients with MASLD, synthesizing current evidence to inform clinical practice and identify areas for future research.

## Review

Methodology

This review was conducted in accordance with the Preferred Reporting Items for Systematic Reviews and Meta-Analyses (PRISMA) guidelines.

Search Strategy

PubMed, Embase, Cochrane Library, and Web of Science databases were searched from January 1, 2015, to August 15, 2025, without applying language restrictions to pick out the full texts of relevant studies. Two authors performed this search. The search strategy incorporated both Medical Subject Headings (MeSH) terms and free-text keywords related to MASLD, NAFLD, sarcopenia, and muscle mass. The following search terms were used in combination with Boolean operators (AND, OR): ("metabolic dysfunction-associated steatotic liver disease" OR "MASLD" OR "nonalcoholic fatty liver disease" OR "NAFLD" OR "metabolic dysfunction-associated steatohepatitis" OR "MASH" OR "nonalcoholic steatohepatitis" OR "NASH" OR "fatty liver disease") AND ("sarcopenia" OR "sarcopenic" OR "muscle wasting" OR "muscle atrophy" OR "skeletal muscle mass" OR "muscle strength" OR "muscle function" OR "appendicular lean mass" OR "skeletal muscle index"). Additional searches were conducted in Google Scholar to identify grey literature, conference abstracts, and unpublished studies. Reference lists of included studies and relevant review articles were manually screened to identify additional eligible studies that might have been missed in the initial database searches. Studies published in languages other than English were translated when necessary.

Study Selection

The study selection process was conducted independently by two reviewers using a standardized approach. Initially, titles and abstracts of all retrieved articles were screened against predefined inclusion and exclusion criteria. Full-text articles of potentially eligible studies were then obtained and assessed for final inclusion. Any disagreements between reviewers were resolved through discussion, and when consensus could not be reached, a third reviewer was consulted. Studies were included if they met the following criteria: (1) cross-sectional, case-control, or cohort studies involving adult patients (≥18 years) with MASLD or NAFLD and (2) studies that compared sarcopenia prevalence between MASLD/NAFLD patients and non-MASLD/NAFLD patients.

Studies were excluded if they (1) included pediatric populations (<18 years); (2) involved patients with other liver diseases (viral hepatitis, alcoholic liver disease, autoimmune liver disease, or drug-induced liver injury); (3) were case reports, case series, editorials, commentaries, or review articles; (4) had insufficient data for analysis; and (5) were duplicate publications.

Data Extraction

Data extraction was performed independently by two reviewers using a standardized data extraction form developed specifically for this study. The following information was systematically extracted from each included study: (1) study characteristics including first author, publication year, country, study design, and sample size; (2) population characteristics including age, sex distribution, body mass index, and comorbidities; (3) sarcopenia measurements method; and (4) prevalence of sarcopenia in MASLD/NAFLD patients and control groups. For studies with multiple publications using the same cohort, the most comprehensive or recent publication was included to avoid duplication.

Quality Assessment

The methodological quality of included studies was assessed using appropriate quality assessment tools based on study design. For cross-sectional studies, the Agency for Healthcare Research and Quality (AHRQ) checklist [16] was used, which evaluates studies across 11 domains, including study population definition, response rate, data collection methods, and statistical analysis. For case-control studies, the Newcastle-Ottawa Scale (NOS) was applied [17], assessing selection of study groups, comparability, and outcome ascertainment. Cohort studies were evaluated using the NOS for cohort studies, examining selection, comparability, and outcome domains.

Two reviewers independently assessed the quality of each study, and disagreements were resolved through discussion. Studies were classified as high quality (low risk of bias), moderate quality (moderate risk of bias), or low quality (high risk of bias) based on their overall assessment scores. The risk of bias assessment considered factors such as representativeness of the study population, validity of diagnostic methods, completeness of outcome data, and potential confounding factors.

Data Analysis

Statistical analyses were performed using RevMan 5.4 (The Cochrane Collaboration, London, England, UK). To compare the prevalence of sarcopenia in patients with and without MASLD/NAFLD, pooled OR with 95% CI were calculated using the Mantel-Haenszel method. Statistical heterogeneity was assessed using the I² statistic and Cochran's Q test. I² values of <25%, 25%-50%, 50%-75%, and >75% were interpreted as low, moderate, substantial, and considerable heterogeneity, respectively. A random-effects model was used irrespective of the heterogeneity to deal with variation among the studies when performing the pooled analysis. Statistical significance was set at p < 0.05 for all analyses.

Results

**Figure 1:**
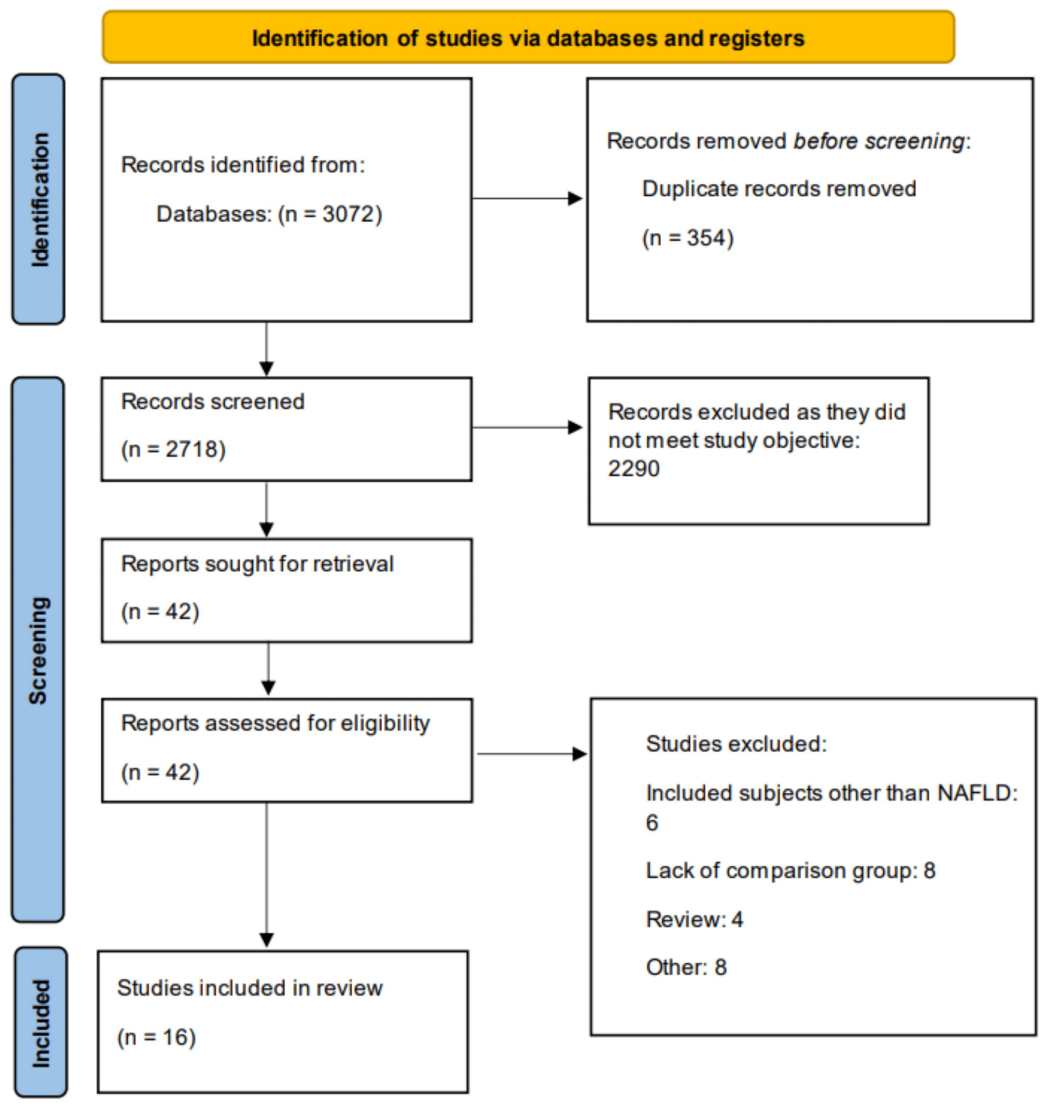
PRISMA flowchart of study selection process

A total of 3,072 articles were identified through database searches. After excluding 354 duplicates, the titles and abstracts of the remaining studies were screened. Subsequently, 42 full-text articles were assessed for eligibility, of which 16 met the inclusion criteria and were incorporated into the meta-analysis. The study selection process is illustrated in the PRISMA flow diagram (Figure 1).

Characteristics of Studies

The characteristics of the included studies are shown in Table 1. A total of 16 studies were included in this review, comprising both cross-sectional and cohort designs and conducted across diverse regions including Korea, China, the United States, Italy, the Netherlands, and the United Kingdom. The sample sizes varied considerably, ranging from 57 to 18,815 participants. Sarcopenia was assessed using different methods, including dual-energy X-ray absorptiometry (DXA), bioelectrical impedance analysis (BIA), computed tomography (CT), and functional tests such as handgrip strength (HGS) and gait speed. The mean age of participants ranged from 23 to 69 years, with a male proportion between 39% and 78%. The average BMI across studies varied from 23.3 to 34.6 kg/m², reflecting differences in population characteristics. Several studies reported the prevalence of comorbidities, with hypertension ranging from 14% to 70% and diabetes from 3% to 32%, although not all studies provided these data. Table 2 presents the quality assessment of the included studies.

**Table 1:** Characteristics of the included studies LMM: low muscle mass, DXA: dual-energy X-ray absorptiometry, HGS: handgrip strength, 6mGS: 6-m gait speed, 4mGS: 4-m gait speed, CT: computed tomography, BIA: bioelectrical impedance analysis, FLI: fatty liver index, MRI-PDFF: magnetic resonance imaging–proton density fat fraction, HSI: hepatic steatosis index, NAFLD: non-alcoholic fatty liver disease, NR: not reported, BMI: body mass index.

Author	Year	Region	Study design	NAFLD/control	Assessment method to detect sarcopenia	Methods used to diagnose NAFLD	Mean age	Male	Mean BMI	Hypertension	Diabetes
Alferink et al. [18]	2019	Netherlands	Cross-sectional	1623/2986	LMM (DXA) + LMS (HGS) + LPP (6mGS)	Ultrasonography	69.3	1982	NR	NR	NR
Choe et al. [19]	2018	Korea	Cross-sectional	716/1112	LMM (CT)	Ultrasonography	55	1121	23.4	730	59
Chung et al. [20]	2019	Korea	Cross-sectional	6298/11242	LMM (BIA)	Ultrasonography	53.2	3431	23.4	892	299
Debroy et al. [21]	2019	Italy	Cross-sectional	57/112	LMM (DXA) + LMS (HGS)	CT	56.9	169	24.6	119	30
Gan et al. [22]	2020	China	Cross-sectional	1088/2448	LMM (DXA) + LMS (HGS)	Ultrasonography	52.8	1016	23.78	1151	220
Golabi et al. [23]	2020	United States	Cohort	1351/3260	LMM (DXA)	US FLI score	46.5	2224	27.7	2261	387
Harring et al. [24]	2023	United States	Cross-sectional	1056/1366	LMM (DXA)	Elastography	38.24	1211	34.61	937	222
Jiang et al. [25]	2021	China	Cross-sectional	301/491	LMM (DXA)	Ultrasonography	64.5	452	27.77	461	792
Kim et al. [26]	2021	United States	Cohort	3773/7292	LMM (BIA)	Ultrasonography	43	5245	27	2325	690
Kim et al. [27]	2024	Korea	Cross-sectional	12327/5827	LMM (BIA)	Ultrasonography	54	11651	24.58	1954	NR
Linge et al. [28]	2021	United Kingdom	Cross-sectional	1204/4122	LMM (DXA) + LMS (HGS)	MRI-PDFF	62.65	2980	26.43	NR	295
Moon et al. [29]	2021	Korea	Cohort	6488/21572	LMM (DXA)	HSI value > 36	50.56	13497	23.99	8310	3163
Park et al. [30]	2020	Korea	Cross-sectional	747/596	LMM (BIA)	Ultrasonography	46.81	747	23.44	NR	NR
Seo et al. [31]	2020	Korea	Cross-sectional	1278/2932	LMM (BIA)	Ultrasonography	57.33	2160	24.72	NR	NR
Wang et al. [32]	2021	China	Cross-sectional	154/424	LMM (DXA) + LMS (HGS) + LPP (4mGS)	Ultrasonography	65.31	92	23.29	NR	NR
Wei et al. [33]	2025	United States	Cohort	664/1167	LMM (DXA)	Elastography	39.41	910	28.95	426	143

**Table 2:** Quality assessment of the included studies For cross-sectional studies: Agency for Healthcare Research and Quality (AHRQ) checklist. For cohort studies: Newcastle-Ottawa Scale. High: Low risk of bias across most domains; well-conducted study with minimal methodological concerns. Moderate-high: Low to moderate risk of bias; generally well-conducted with minor limitations. Moderate: Moderate risk of bias; acceptable methodology but with some notable limitations. Low: High risk of bias; significant methodological concerns that may affect the validity of results.

Author	Study design	Overall
Alferink et al. [18]	Cross-sectional	High
Choe et al. [19]	Cross-sectional	High
Chung et al. [20]	Cross-sectional	High
Debroy et al. [21]	Cross-sectional	Moderate-high
Gan et al. [22]	Cross-sectional	High
Golabi et al. [23]	Cohort	High
Harring et al. [24]	Cross-sectional	High
Jiang et al. [25]	Cross-sectional	Moderate-high
Kim et al. [26]	Cohort	Moderate
Kim et al. [27]	Cross-sectional	High
Linge et al. [28]	Cross-sectional	Moderate
Moon et al. [29]	Cohort	Moderate
Park et al. [30]	Cross-sectional	Moderate
Seo et al. [31]	Cross-sectional	Low
Wang et al. [32]	Cross-sectional	Moderate
Wei et al. [33]	Cohort	High

Results of the Meta-analysis

**Figure 2:**
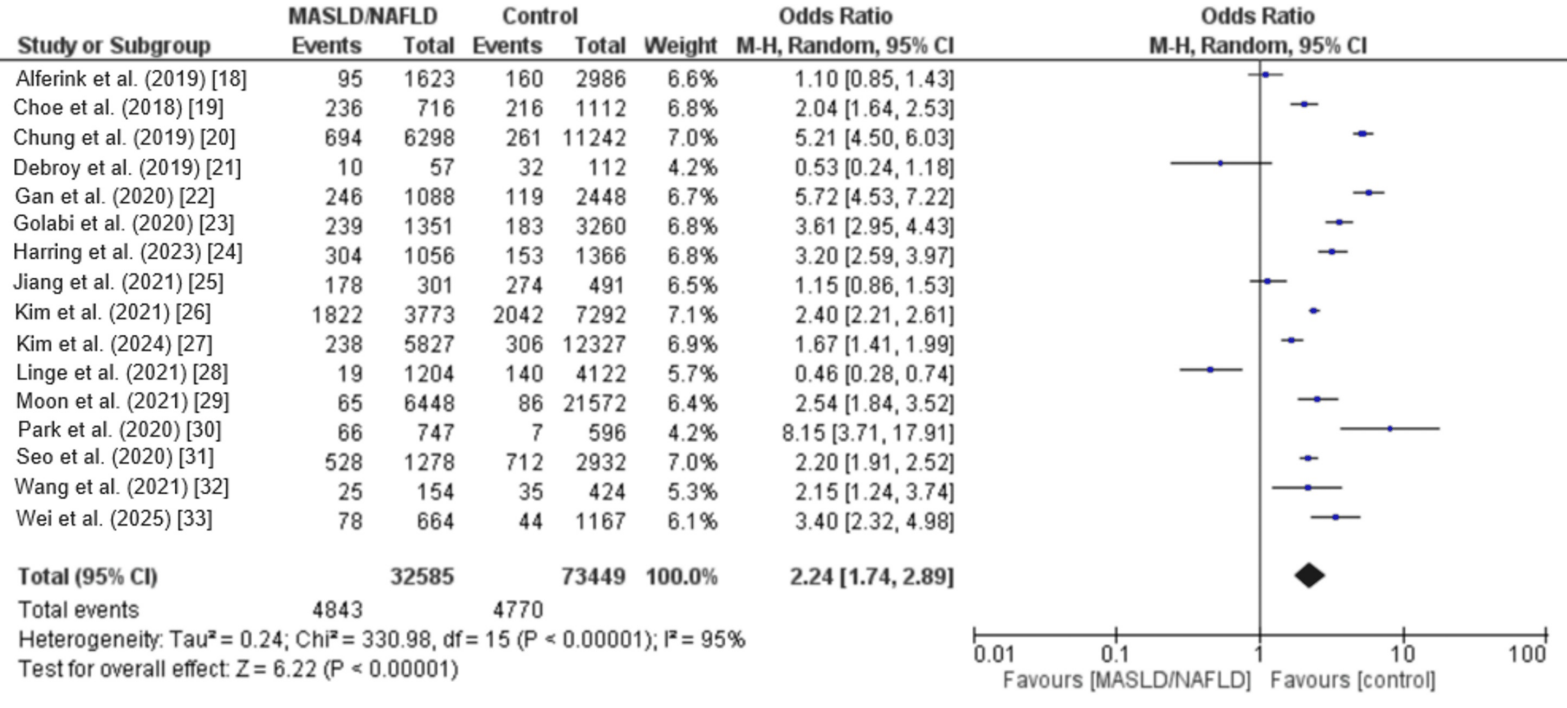
Comparison of the prevalence of sarcopenia between the two groups Source: [18-33].

Figure 2 presents the pooled analysis showing the effect of MAFLD on the risk of sarcopenia. The prevalence of sarcopenia was 14.86% in MAFLD subjects and 6.49% in non-MAFLD subjects. The pooled analysis showed that the odds of sarcopenia in subjects with MAFLD was significantly higher compared to the control subjects (OR: 2.24, 95% CI: 1.74 to 2.89). High heterogeneity was reported among the study results (I-Square: 95%).

Subgroup Analyses

Table 3 shows the subgroup analyses. In the subgroup analyses, the association between sarcopenia and NAFLD remained significant across most categories, although the magnitude varied. By study design, cohort studies showed a stronger association (OR = 2.90, 95% CI: 2.27-3.72; I² = 81%) compared with cross-sectional studies (OR = 2.02, 95% CI: 1.39-2.94; I² = 97%). When stratified by region, the association was highest in studies conducted in Korea (OR = 3.10, 95% CI: 1.76-5.45; I² = 97%) and the United States (OR = 3.06, 95% CI: 2.40-3.90; I² = 87%), whereas studies from China showed a weaker and non-significant association (OR = 2.43, 95% CI: 0.78-7.58; I² = 97%). Subgrouping by mean age demonstrated a stronger association in populations under 50 years (OR = 3.33, 95% CI: 2.55-4.35; I² = 85%) compared to those aged 50 years or older (OR = 1.79, 95% CI: 1.20-2.65; I² = 97%). Finally, studies published from 2020 onward reported a higher pooled effect (OR = 2.42, 95% CI: 1.78-3.30; I² = 95%) than those published before 2020 (OR = 1.85, 95% CI: 1.07-3.20; I² = 96%).

**Table 3:** Subgroup analyses

Variable	Groups	OR (95% CI)	I²
Study design	Cohort	2.90 (2.27, 3.72)	81%
	Cross-sectional	2.02 (1.39, 2.94)	97%
Region	United States	3.06 (2.40, 3.90)	87%
	Korea	3.10 (1.76, 5.45)	97%
	China	2.43 (0.78, 7.58)	97%
Mean age	<50	3.33 (2.55, 4.35)	85%
	>50	1.79 (1.20, 2.65)	97%
Publication year	Before 2020	1.85 (1.07, 3.20)	96%
	2020 onward	2.42 (1.78, 3.30)	95%

Discussion

The objective of this meta-analysis was to evaluate the prevalence of sarcopenia in individuals with MASLD compared to those without the condition. The findings demonstrated a significant association between MASLD and sarcopenia, with a higher prevalence observed in patients with MASLD (14.86%) than in control subjects (6.49%). Similarly, a recent meta-analysis by Li et al. reported an overall pooled prevalence of sarcopenia of 23.5% among patients with MASLD [15].

The bidirectional relationship between MASLD and sarcopenia is supported by several pathophysiological mechanisms. Skeletal muscle serves as the primary site for glucose disposal and plays a crucial role in maintaining insulin sensitivity [34]. Loss of muscle mass and function contributes to insulin resistance, promoting hepatic lipogenesis and steatosis development [35]. Conversely, MASLD-associated chronic inflammation, characterized by elevated pro-inflammatory cytokines such as tumor necrosis factor-α and interleukin-6, can accelerate muscle protein breakdown and impair muscle regeneration [36]. Additionally, altered hepatokine secretion in MASLD patients may disrupt muscle metabolism and contribute to sarcopenia development [37]. Alterations in hormone levels, including testosterone and estrogen, have been reported to influence muscle structure and function, although their precise effects remain a topic of debate [38,39]. For instance, one study [38] found that reduced testosterone levels were linked to lower muscle mass, which in turn promoted hepatic steatosis by affecting fat synthesis and secretion in mice fed a high-fat diet. In contrast, another study [39] reported that baseline testosterone levels did not independently impact the progression or regression of MASLD.

The substantial heterogeneity observed in our meta-analysis (I² = 95%) reflects the diverse methodological approaches employed across included studies. This heterogeneity stems from several sources, including variations in sarcopenia diagnostic criteria, MASLD assessment methods, and population characteristics. Studies employed different sarcopenia assessment techniques, ranging from DXA and BIA to CT imaging and functional tests such as HGS. Each method has distinct advantages and limitations, with DXA considered the gold standard for muscle mass assessment, while functional tests provide information about muscle strength and physical performance [40,41].

The diagnostic criteria for MASLD also varied across studies, with some using the traditional NAFLD definition based on exclusion criteria, while others employed the newer MASLD criteria emphasizing positive cardiometabolic risk factors [42]. This transition in nomenclature and diagnostic criteria may partially explain the temporal trend observed in our publication year subgroup analysis, where studies published from 2020 onward showed higher effect sizes. Despite the high heterogeneity, the consistent direction of the association across subgroups and the statistical significance of the pooled estimate support the robustness of our findings. The use of random-effects models appropriately accounts for between-study variability, providing conservative estimates of the association.

While previous systematic review and meta-analyses assessed the association between sarcopenia and MASLD [1,42], they comprised limited cross-sectional studies lacking a thorough subgroup analysis. This report conducted comprehensive research and also included recently conducted studies.

The strong association between MASLD and sarcopenia has several important clinical implications. First, it suggests that MASLD patients should undergo routine screening for sarcopenia, particularly those with advanced disease or multiple metabolic comorbidities. Early identification of sarcopenia in MASLD patients could facilitate timely interventions to prevent further loss of muscle mass and associated complications [43].

Second, the bidirectional nature of the MASLD-sarcopenia relationship suggests that interventions targeting muscle health may also benefit liver outcomes. Resistance exercise training and nutritional interventions aimed at preserving or increasing muscle mass could potentially improve both sarcopenia and MASLD outcomes [44]. The recent approval of resmetirom for MASH treatment provides new therapeutic options, though its effects on muscle health remain to be determined [45,46].

Several limitations should be acknowledged when interpreting our findings. First, the cross-sectional nature of most included studies limits our ability to establish causality or determine the temporal sequence of MASLD and sarcopenia development. Second, the substantial heterogeneity between studies, while addressed through subgroup analyses, may limit the generalizability of our findings. The variation in sarcopenia diagnostic criteria across studies may have influenced our results, as different methods may identify distinct populations with varying degrees of muscle impairment. Finally, most included studies were conducted in specific geographic regions, potentially limiting the global applicability of our findings.

## Conclusions

This systematic review and meta-analysis demonstrates a significant association between sarcopenia and MASLD across diverse populations and study designs. Sarcopenia prevalence was consistently higher in MASLD patients compared to controls, with this relationship persisting across different measurement methodologies and geographic regions despite substantial study heterogeneity. Clinical implications are substantial. MASLD patients should undergo routine sarcopenia screening, particularly those with advanced disease. Early identification could facilitate timely interventions, including resistance exercise training and nutritional support that may benefit both conditions simultaneously. Future research should focus on longitudinal studies to establish causality, standardized diagnostic criteria to reduce methodological heterogeneity, and investigation of targeted therapeutic interventions addressing both hepatic and muscular manifestations of this complex relationship.
